# Correction: Mitochondrial genome sequence of *Phytophthora sansomeana* and comparative analysis of *Phytophthora* mitochondrial genomes

**DOI:** 10.1371/journal.pone.0236076

**Published:** 2020-07-09

**Authors:** The PLOS Staff

Fig 3 is incorrect. The authors have provided a corrected version here. The publisher apologizes for the error.

**Fig 3:**
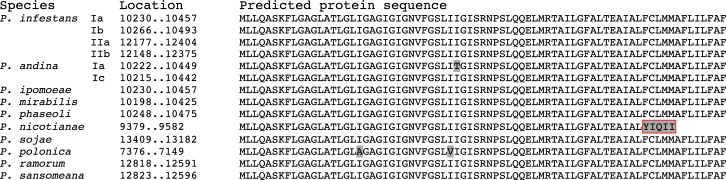
Deduced protein sequences of *atp9* gene in the mitochondrial genomes of 10 *Phytophthora* species. Amino acids differ from the consensus are shaded in grey. A deletion at the 3’ end of this gene in *P*. *nicotianae* results in the replacement of the last 13 amino acids with five non-homologous ones (boxed in red).
